# Supplementary material for: Dynamic 1D Search and Processive Nucleosome Translocations by RSC and ISW2 Chromatin Remodelers
Source: bioRxiv. 2024 Jan 15:2023.06.13.544671. Preprint. [Version 2] doi: 10.1101/2023.06.13.544671 (PMC10827135; doi:10.1101/2023.06.13.544671)
Supplement: Supplement 1 [file NIHPP2023.06.13.544671v2-supplement-1.pdf]

bioRxiv preprint doi: https://doi.org/10.1101/2023.06.13.544671; this version posted January 15, 2024. The copyright holder for this preprint (which was not certified by peer review) is the author/funder, who has granted bioRxiv a license to display the preprint in perpetuity. It is made available under aCC-BY 4.0 International license.

Supplemental Figures

Figure 1 – figure supplement 1. RSC and ISW2 diffusion analysis under varied conditions. (A) Schematic of the microfluidics chamber used, with four solution compartments (1-3 separated by laminar flow, 4 as reservoir). (B) Construct map displaying RSC and ISW2 labeling via 3x-FLAG tag and HaloTag at N-terminus of ATPase subunits. (C) SDS-PAGE gel showing JFX650-labeled purified RSC (left) and ISW2 (right) complexes, with protein visualization using Flamingo dye. (D) Proportion of dCas9, RSC, and ISW2 traces in three diffusion categories under baseline conditions (50 mM KCl, no ATP). K-means clustering identifies five distinct diffusion behaviors (C1-5), with percentage of traces in each group. (E, G) Scatter violin plots show mean diffusion coefficients for 'non-diffusive', low, and high diffusion groups. Mann-Whitney tests compare RSC and ISW2 distributions in the high-diffusive category. Median D_{coef} values and 95% confidence intervals are represented. (F, H) Proportion of traces in the three diffusion categories; K-means clustering identifies five diffusion classes (C1-5) with percentage of each group printed. (E-F) Effects of salt on diffusion: 50 mM, 100 mM, and 200 mM KCl for RSC, and 50 mM, 75 mM, and 100 mM KCl for ISW2 (no binding detected at 200 mM KCl for ISW2). (G-H) Effects of nucleotide on diffusion: no ATP, 1 mM ATP, and 1 mM ATPγS.

Figure 1 – figure supplement 2. Halo-tagged remodeler functional validation, labeling and purification. (A-B) Native PAGE analysis of nucleosome sliding by HaloTagged RSC (A) and ISW2 (B). RSC shifts centered nucleosomes (43N43; Cy5-DNA) to end positions, while ISW2 moves end-positioned nucleosomes (80N3; Cy5-DNA, Cy3-H2A) to a centered position. (C) Assessment of protein labeling efficiency using fluorescence intensity of the fluorophore label; protein resolved via SDS-PAGE gel and scanned using a Typhoon imager. (D) Flow-chart of protein labeling and purification. (E-F) Fractions from glycerol gradient purification of labeled protein run on SDS-PAGE gels and imaged for fluorophore label and total protein content using a Typhoon imager for (E) RSC-JFX650 and (F) ISW2-JFX650 preparations.

Figure 2 – figure supplement 1. Detection of remodeler-remodeler interactions and dwell-time estimation. (A) Simulated images illustrating diffusive molecules and camera noise, with distinct molecule types shown in different colors. (B) Single-molecule trajectories obtained from the analysis of images in (A). (C) Plot of the distance between the two molecules (blue) with interaction length indicated by gray (longer-lived) or red (short-lived) shaded regions. Short interactions are defined as lasting less than 5 frames and showing higher distance changes compared to the mean displacement. (D-F) Magnified versions of (A-C) for improved visualization.

Figure 3 – figure supplement 1. Remodeler-nucleosome interactions and nucleosome sliding assays. (A) Electrophoretic mobility shift assay (EMSA) of lambda nucleosome arrays with increasing octamer concentration. The asterisk indicates a condition that produced ~10 nucleosomes per array. (B) Representative kymograph of remodeler diffusion on a nucleosome array, accompanied by a schematic for interpretation. The green laser is pulsed to extend the fluorophore's lifetime. Remodeler-nucleosome interactions are indicated within the kymograph, with additional examples shown in panels D-G. (C-D) Examples of RSC-nucleosome interactions, which share visual similarities with ISW2-nucleosome interactions. (C) Remodelers colliding with nucleosomes without extended interaction. (D) Prolonged colocalization events without translocation. (E) Bypass events where the remodeler passes a nucleosome. (F) Observable nucleosome translocation events, with the starting position marked as a reference.

bioRxiv preprint doi: https://doi.org/10.1101/2023.06.13.544671; this version posted January 15, 2024. The copyright holder for this preprint (which was not certified by peer review) is the author/funder, who has granted bioRxiv a license to display the preprint in perpetuity. It is made available under aCC-BY 4.0 International license.

Figure 3 – figure supplement 2. Nucleosome translocation durations and half-lives. (A-B) 1-Cummulative Distribution Function (CDF) survival curves of nucleosome translocation events for (A) RSC and (B) ISW2 in the presence of 1 mM ATP. Half-lives from single-exponential fitting printed in graph.

Figure 5 – figure supplement 1. Various translocation scenarios support the push-pull model for RSC-ISW2. (A-B) Pie-charts of “pushing” and “pulling” counts for RSC (A) and ISW2 (B); various translocation scenarios are displayed separately within the pie chart. The identities of the wedges are indicated with a legend shown alongside schematics of the various scenarios (C-D), where (C) shows scenarios that support the push model whereas (D) shows scenarios that support the pull model. A key to interpret the schematic is displayed below.

bioRxiv preprint doi: https://doi.org/10.1101/2023.06.13.544671; this version posted January 15, 2024. The copyright holder for this preprint (which was not certified by peer review) is the author/funder, who has granted bioRxiv a license to display the preprint in perpetuity. It is made available under aCC-BY 4.0 International license.

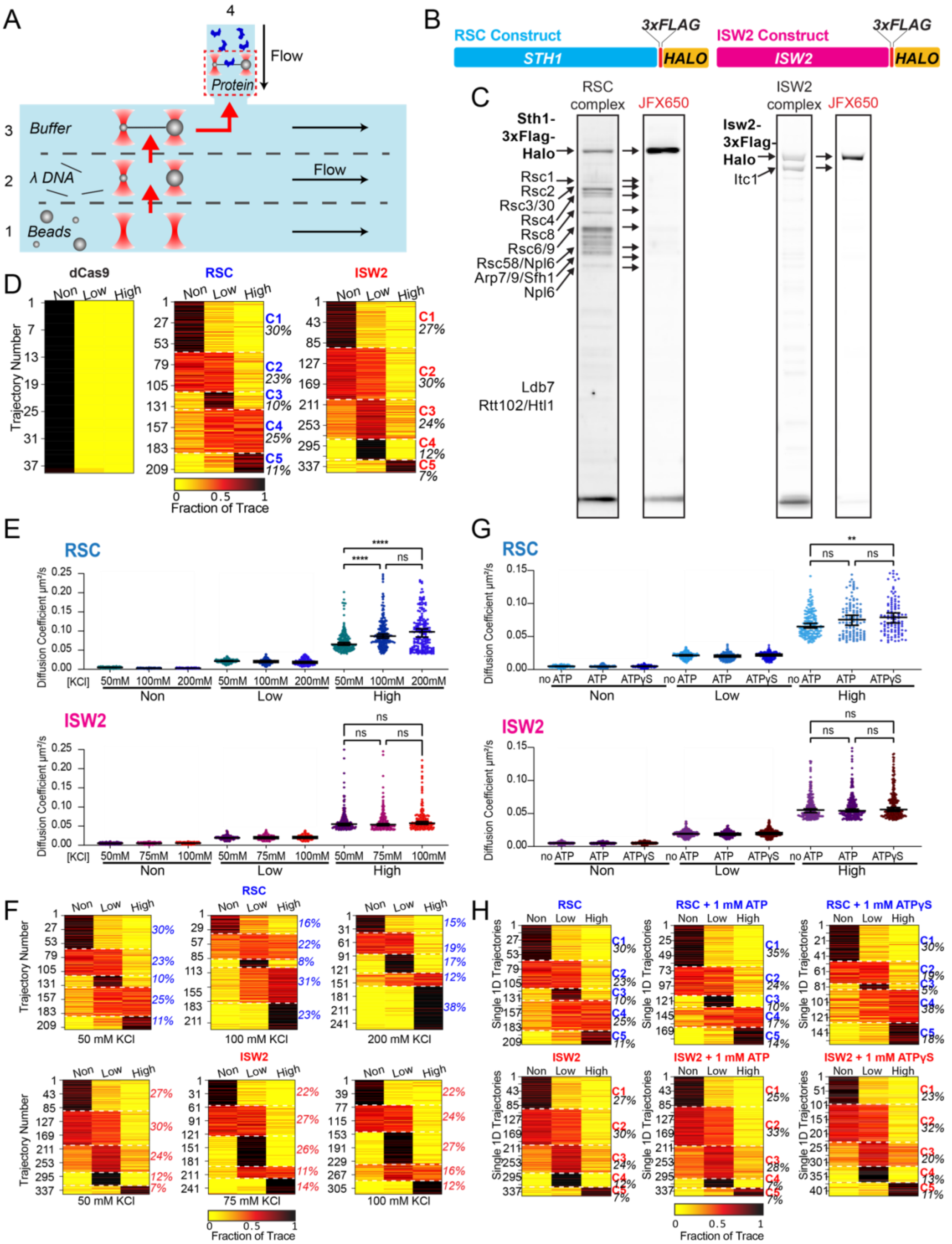

Figure 1 - figure supplement 1

bioRxiv preprint doi: https://doi.org/10.1101/2023.06.13.544671; this version posted January 15, 2024. The copyright holder for this preprint (which was not certified by peer review) is the author/funder, who has granted bioRxiv a license to display the preprint in perpetuity. It is made available under aCC-BY 4.0 International license.

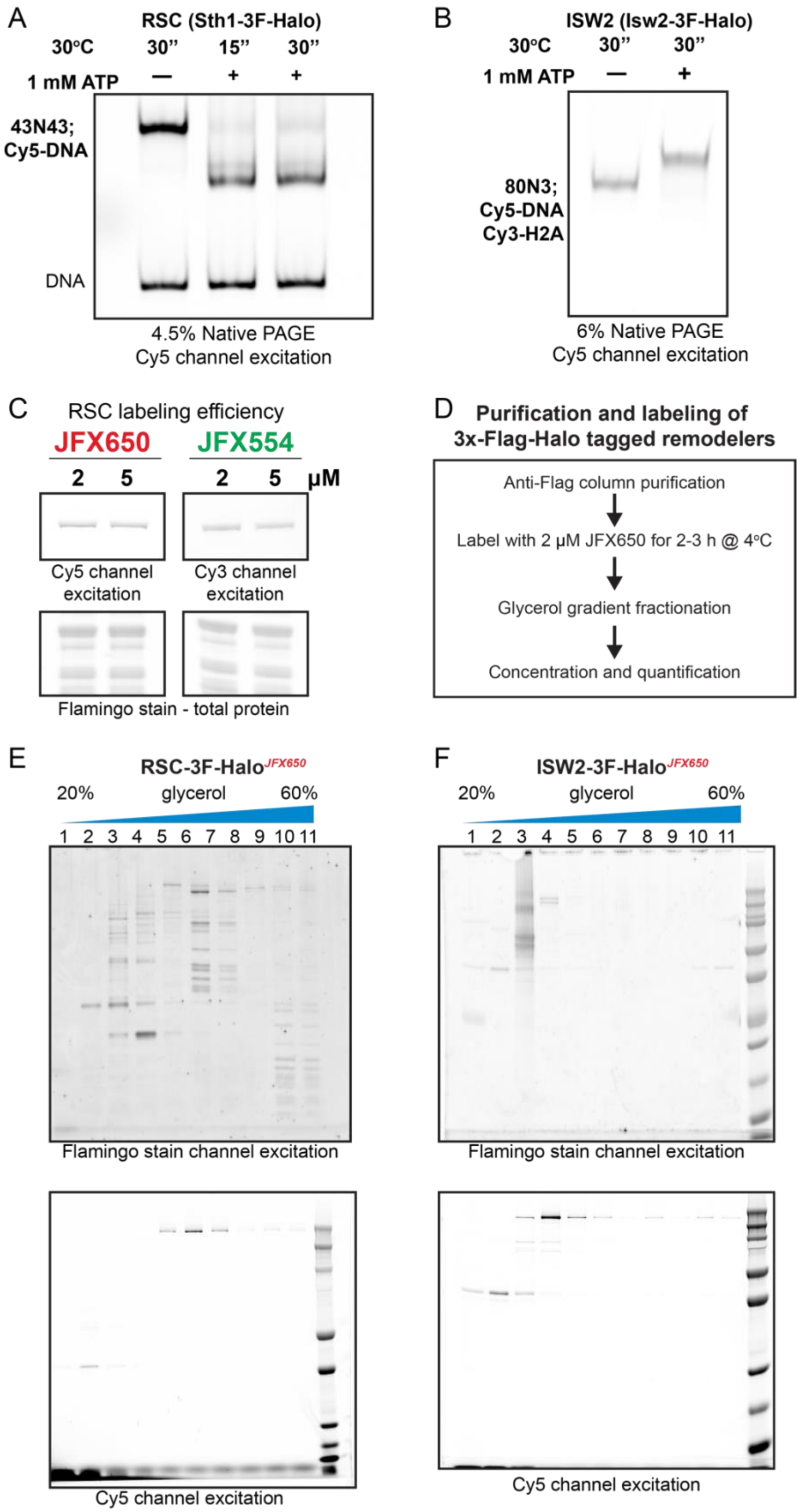

bioRxiv preprint doi: https://doi.org/10.1101/2023.06.13.544671; this version posted January 15, 2024. The copyright holder for this preprint (which was not certified by peer review) is the author/funder, who has granted bioRxiv a license to display the preprint in perpetuity. It is made available under aCC-BY 4.0 International license.

Figure 1 – figure supplement 2

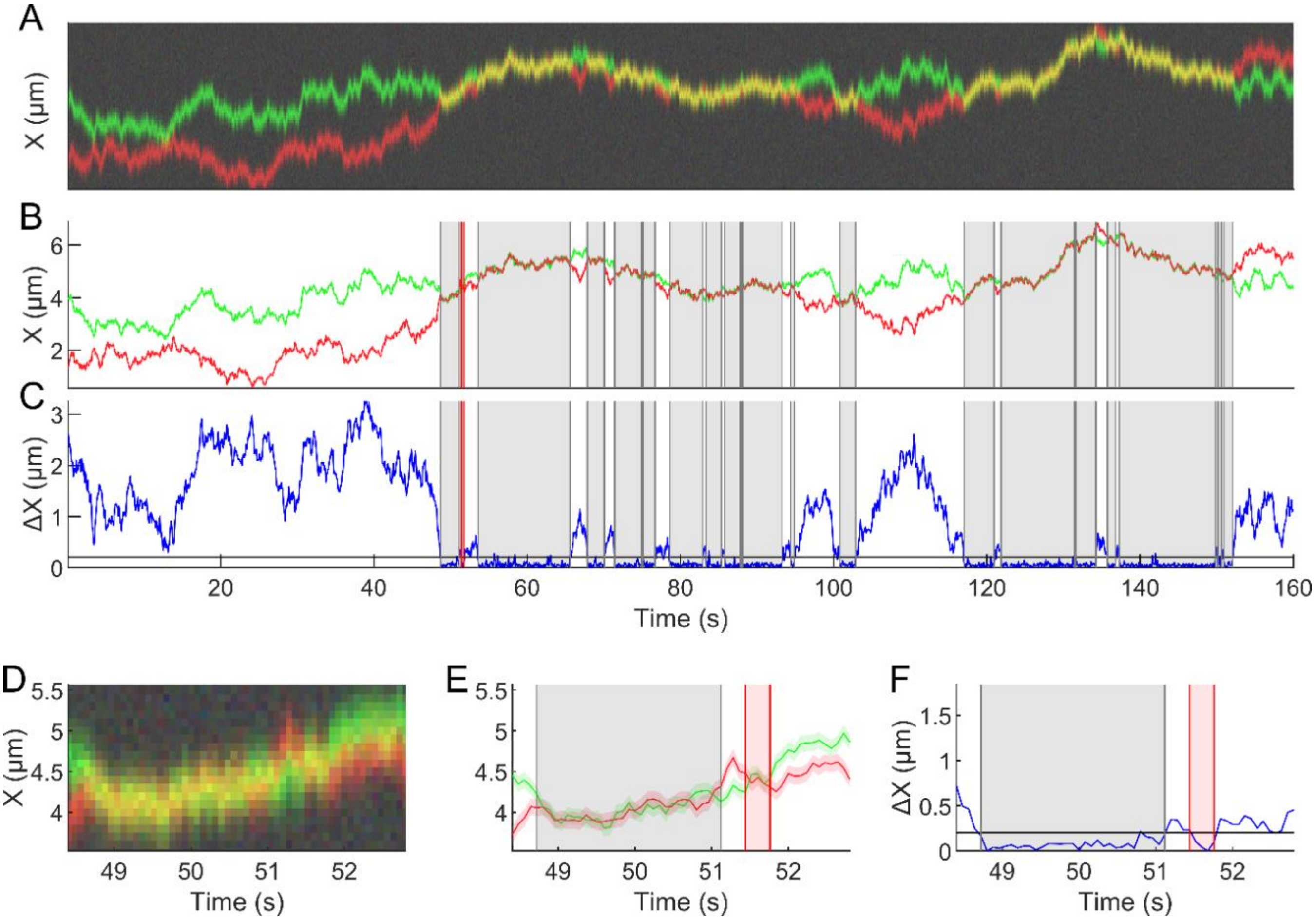

Figure 2 – figure supplement 1

bioRxiv preprint doi: https://doi.org/10.1101/2023.06.13.544671; this version posted January 15, 2024. The copyright holder for this preprint (which was not certified by peer review) is the author/funder, who has granted bioRxiv a license to display the preprint in perpetuity. It is made available under aCC-BY 4.0 International license.

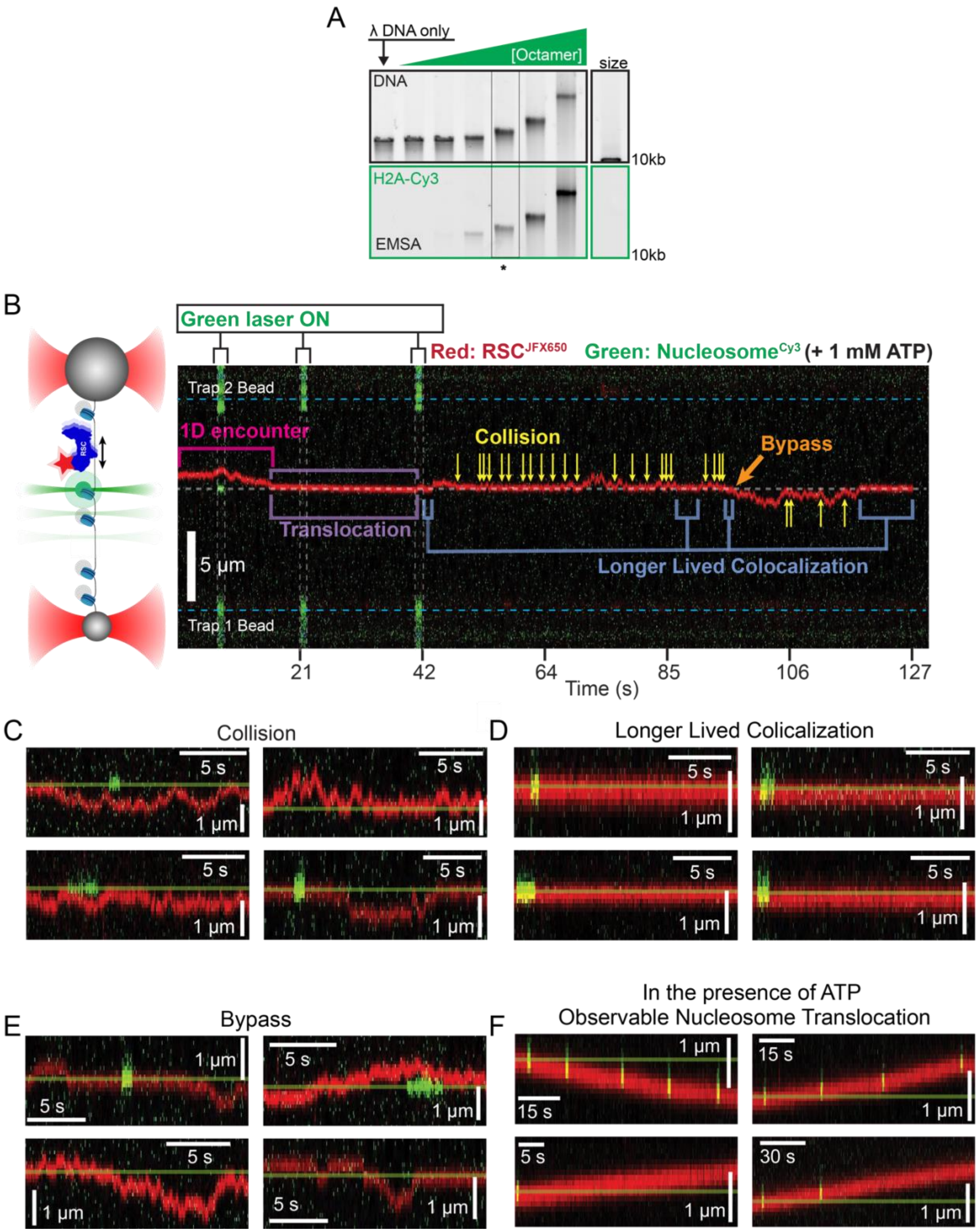

Figure 3 – figure supplement 1

bioRxiv preprint doi: https://doi.org/10.1101/2023.06.13.544671; this version posted January 15, 2024. The copyright holder for this preprint (which was not certified by peer review) is the author/funder, who has granted bioRxiv a license to display the preprint in perpetuity. It is made available under aCC-BY 4.0 International license.

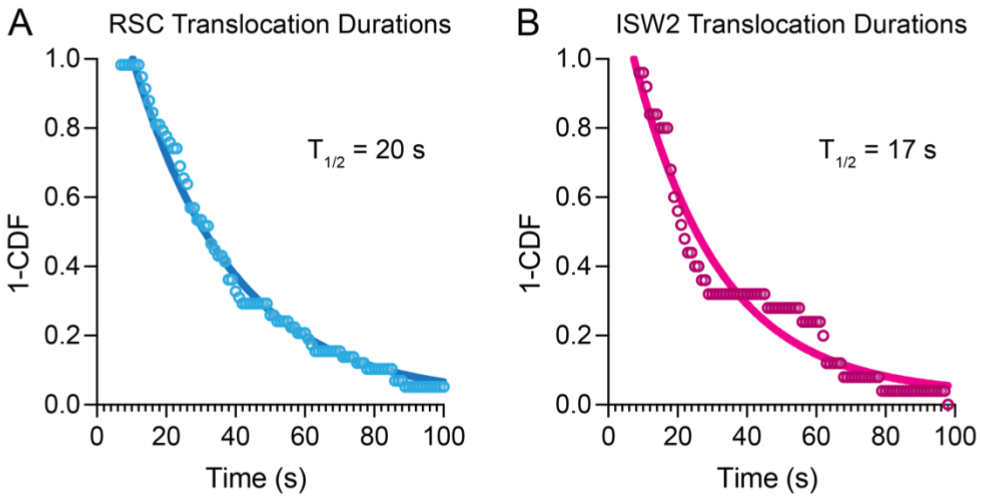

Figure 3 – figure supplement 2

bioRxiv preprint doi: https://doi.org/10.1101/2023.06.13.544671; this version posted January 15, 2024. The copyright holder for this preprint (which was not certified by peer review) is the author/funder, who has granted bioRxiv a license to display the preprint in perpetuity. It is made available under aCC-BY 4.0 International license.

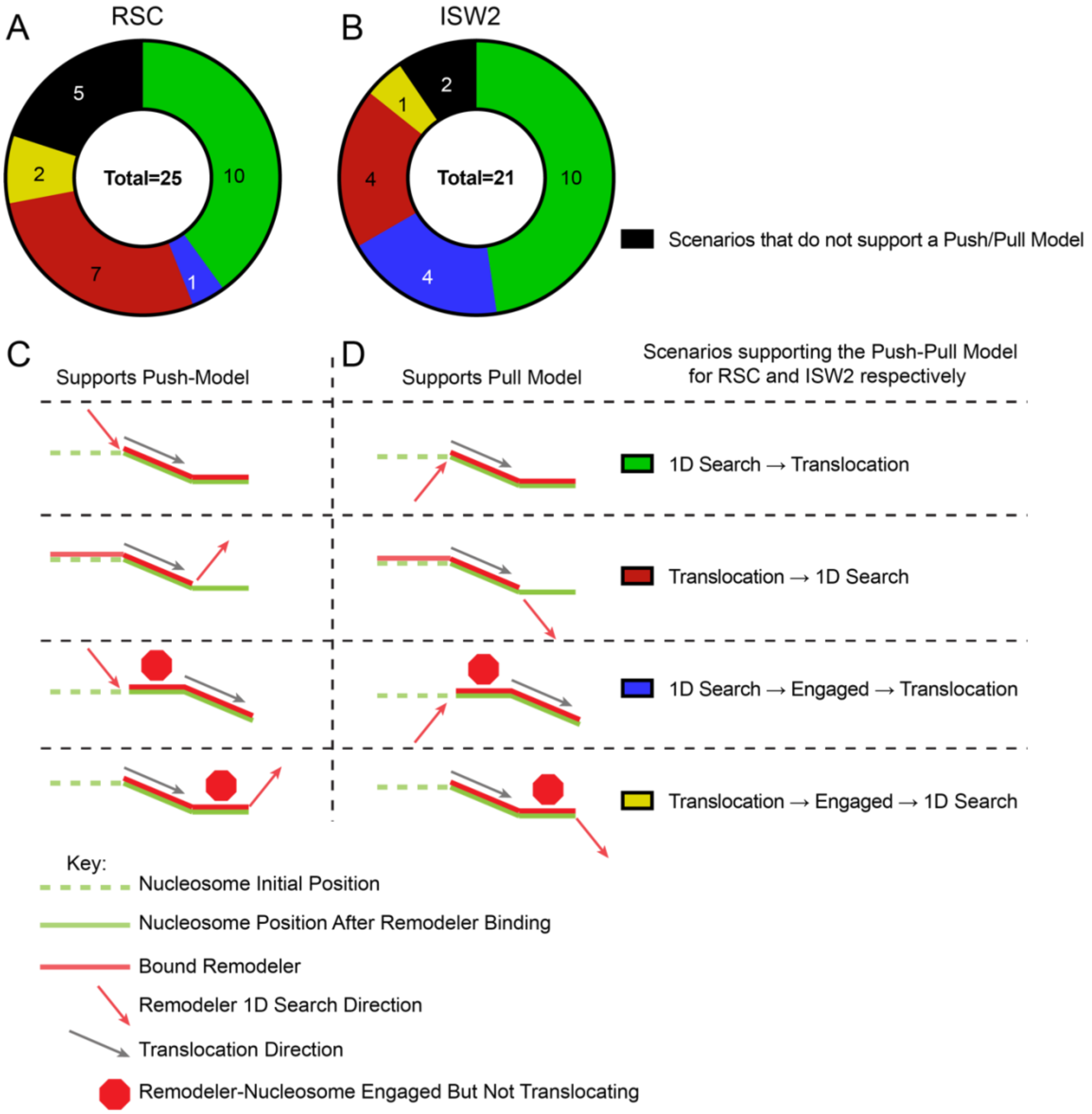

Figure 5 – figure supplement 1
